# Bioelectrical Impedance and GLIM Criteria Identify Early Nutritional Deterioration and Mortality in Acute Leukemia Patients Undergoing Chemotherapy

**DOI:** 10.3390/nu18030374

**Published:** 2026-01-23

**Authors:** Lara Dalla Rovere, María José Tapia Guerrero, Viyey K. Doulatram-Gamgaram, María Garcia-Olivares, Belén del Arco-Romualdo, Montserrat Gonzalo-Marín, María Rosario Vallejo Mora, Daniel Barrios Decoud, Carola Díaz Aizpún, Francisco José Sánchez-Torralvo, Cristina Herola-Cobos, Carmen Hardy-Añón, Agustín Hernandez-Sanchez, José Manuel García-Almeida, Gabriel Olveira

**Affiliations:** 1 Department of Endocrinology and Nutrition, Quironsalud Malaga Hospital, 29004 Málaga, Spain; lara.dalla@quironsalud.es (L.D.R.); cristinaherolanutricion@gmail.com (C.H.-C.); carmenhardy018@gmail.com (C.H.-A.); jgarciaalmeida@gmail.com (J.M.G.-A.); 2 Department of Medicine and Dermatology, Málaga University, 29010 Málaga, Spain; gabrielm.olveira.sspa@juntadeandalucia.es; 3 Endocrinology and Nutrition Department, Hospital Regional Universitario Málaga, IBIMA BIONAND Platform, Malaga Biomedical Research Institute, 29590 Málaga, Spain; mjtapiague@gmail.com (M.J.T.G.); bdromualdo@gmail.com (B.d.A.-R.); montsegonzalo@yahoo.es (M.G.-M.); rosariovallejomora@hotmail.com (M.R.V.M.); fjstsol@gmail.com (F.J.S.-T.); 4 CIBERDEM, Carlos III Health Institute (ISCIII), 28031 Madrid, Spain; 5 Endocrinology and Nutrition Department, Hospital Universitario Virgen de la Victoria Málaga, IBIMA BIONAND Platform, Malaga Biomedical Research Institute, 29590 Málaga, Spain; 6 Hematology Department, Hospital Regional Universitario Málaga, 29590 Málaga, Spain; danibarrios7@gmail.com (D.B.D.); carola.diazaizpun@gmail.com (C.D.A.); 7 Department of Haematology, Quironsalud Malaga Hospital, 29004 Málaga, Spain; agustin.hernandez@quironsalud.es; 8 Department of Endocrinology and Nutrition, Virgen de la Victoria University Hospital, 29010 Málaga, Spain; 9 CIBEROBN, Carlos III Health Institute (ISCIII), 28031 Madrid, Spain

**Keywords:** acute leukemia, malnutrition, morphofunctional assessment, phase angle, bioelectrical impedance, handgrip strength, muscle ultrasound, GLIM criteria, body composition

## Abstract

Background/Objectives: Malnutrition is highly prevalent in patients with acute leukemia and is frequently underrecognized at diagnosis. Traditional screening tools based on anthropometry often fail to identify early nutritional deterioration. This study aimed to evaluate the prognostic utility of a comprehensive morphofunctional assessment—including bioelectrical impedance vector analysis (BIVA), handgrip strength (HGS), and muscle ultrasound—conducted at diagnosis and after induction therapy, to evaluate the prognostic association with 12-month mortality. Methods: In this prospective cohort study, 52 adult patients with newly diagnosed acute leukemia were enrolled between November 2022 and November 2024 at two tertiary hospitals in Málaga, Spain. Nutritional status was determined using GLIM criteria. Morphofunctional assessment included BIVA-derived phase angle (PhA), HGS via dynamometry, and rectus femoris ultrasound. A second evaluation was performed prior to haematopoietic stem cell transplantation. Mortality at 12 months was the primary outcome. Logistic regression and ROC analysis were used to assess prognostic associations. Results: At baseline, 65.4% of patients were classified as malnourished. After three months, patients showed significant declines in PhA (−0.55°, *p* < 0.001), body cell mass (−3.15 kg, *p* < 0.01), skeletal muscle mass (−1.66 kg, *p* < 0.01), and rectus femoris cross-sectional area (−0.36 cm^2^, *p* = 0.011). Baseline malnutrition (OR = 6.88; 95% CI: 1.17–40.38; *p* = 0.033) and PhA decline ≥ 0.90° were both independently associated with higher 12-month mortality. Conclusions: Early morphofunctional assessment using GLIM criteria, BIVA, and muscle ultrasound identifies patients at nutritional and functional risk. PhA decline during treatment was associated with higher 12-month mortality, supporting the need for early, personalized nutritional intervention in leukemia care.

## 1. Introduction

Acute leukemias, including acute lymphoblastic leukemia (ALL) and acute myeloid leukemia (AML), are aggressive hematopoietic neoplasms characterized by the uncontrolled proliferation of immature precursor cells. Together, they account for a substantial proportion of leukemia-related morbidity and mortality worldwide, with ALL being the most common leukemia in children and AML predominating in adults, particularly those over 60 years of age. Treatment depends on the type of leukemia but generally involves chemotherapy and, in selected cases, hematopoietic stem cell transplantation (HSCT) [1,2]. These therapeutic modalities are associated with metabolic changes and systemic inflammatory responses, which may worsen nutritional and functional deterioration from the earliest stages of care. During this early treatment phase, patients are highly vulnerable to disease-related malnutrition (DRM) and functional decline, which may substantially impair clinical outcomes [3].

Despite its clinical relevance, malnutrition remains underdiagnosed in hematological patients, particularly before therapy begins. Traditional screening tools—based primarily on anthropometric criteria such as weight loss or body mass index (BMI)—fail to identify early or subclinical alterations in body composition [4]. Recent advances in nutritional assessment recommend a morphofunctional approach [5], integrating structural and functional markers to more precisely capture changes in nutritional and muscular status. This includes bioelectrical impedance vector analysis (BIVA), handgrip strength (HGS), and nutritional ultrasound, in accordance with updated frameworks such as the Global Leadership Initiative on Malnutrition (GLIM) and the European consensus on sarcopenia [6].

Among these tools, the phase angle (PhA) has emerged as a reliable biomarker of cellular integrity and prognosis [7]. Lower baseline PhA and early declines during treatment have been consistently associated with increased mortality, prolonged hospitalization, and treatment intolerance in oncology settings [8,9]. Similarly, declines in muscle cross-sectional area and strength reflect early sarcopenic changes that may represent the initial manifestation of cancer-associated cachexia [10,11].

In this context, the early identification of malnutrition and functional decline is critical. However, most studies in leukemia populations assess nutritional status late in the treatment course or exclusively in transplant candidates, limiting their ability to inform early intervention strategies [12,13]. To address this gap, the present study prospectively evaluated patients with newly diagnosed acute leukemia using a comprehensive morphofunctional assessment prior to chemotherapy initiation and again before HSCT.

The aim was to characterize baseline nutritional status, monitor early changes in muscle mass and cellularity, and determine the prognostic relevance of GLIM-defined malnutrition and PhA dynamics for 12-month mortality.

## 2. Materials and Methods

### 2.1. Study Design and Patients Included in the Study

A prospective study was conducted, including patients diagnosed with acute leukemia at the Regional University Hospital of Málaga and Hospital Quirón Salud of Málaga between November 2022 and November 2024. Patients aged 18 years or older with a confirmed diagnosis of acute leukemia who were scheduled to receive treatment—chemotherapy or other therapeutic modalities—within the month following the nutritional assessment were eligible. Those younger than 18 years or pregnant were excluded. Data collected included the type of acute leukemia (lymphoid, myeloid, or undifferentiated) and the treatment received (intensive chemotherapy, hypomethylating agents, or BCL2 inhibitors).

Longitudinal analyses were conducted in the 52 patients who completed both the baseline and follow-up morphofunctional assessments. The second evaluation was performed after completion of induction chemotherapy and prior to HSCT, with a median interval of approximately three months. Patients with missing follow-up data (n = 17) were excluded from longitudinal analyses, and no imputation was performed.

A flow chart diagram shows the patient selection process for our study (Supplementary Figure S1).

### 2.2. Nutritional Assessment

All patients were assessed during their hospital admission in the Hematology Department prior to treatment initiation. Malnutrition was diagnosed using the Global Leadership Initiative on Malnutrition (GLIM) criteria [14], which require the simultaneous presence of at least one phenotypic and one etiologic criterion to establish a diagnosis. Malnutrition was diagnosed according to the GLIM criteria, requiring the presence of at least one phenotypic and one etiologic criterion. Phenotypic criteria included unintentional weight loss, low BMI, or evidence of muscle impairment. Given the potential confounding effects of fluid shifts on body composition techniques in patients with acute leukemia, reduced muscle status was operationalized using handgrip strength as a functional surrogate, applying sex-specific cut-off points according to the European Working Group on Sarcopenia in Older People (EWGSOP) consensus. Etiologic criteria included reduced food intake and disease-related inflammation related to acute leukemia. Importantly, BIVA-derived parameters, muscle ultrasound measurements, and PhA were not incorporated into the GLIM-based diagnosis of malnutrition but were analyzed separately as independent morphofunctional markers for prognostic evaluation.

Patients who met malnutrition criteria were prescribed a high-protein oral nutritional supplement. In addition to supplementation, personalized high-calorie and high-protein dietary recommendations and an adapted exercise plan focused on muscle strengthening were provided by a trained dietitian, as previously described in detail. Gastrointestinal symptoms potentially affecting intake (e.g., nausea, dysphagia, vomiting, diarrhea) were systematically recorded. Furthermore, a second complete nutritional assessment was performed after completion of induction treatment and prior to hematopoietic stem cell transplantation to monitor changes in nutritional status.

### 2.3. Morphofunctional Assessment

All morphofunctional assessments were conducted by dietitians trained in bioimpedance and nutritional ultrasound, using standardized protocols.

#### 2.3.1. Bioelectrical Impedance Vectoral Analysis

BIVA was performed using a 50 kHz phase-sensitive impedance analyzer (Whole Body Bioimpedance Vector Analyzer, Nutrilab^®^, AKERN Srl, Florence, Italy). After 5 min in the supine position, electrodes were positioned on the right hand and foot, and measurements were obtained. Parameters recorded included phase angle (PhA, °), standardized phase angle (SPhA), body cell mass (BCM, kg), body cell mass index (BCMI, kg/m^2^), fat mass (FM, kg), fat mass index (FMI, kg/m^2^), fat-free mass index (FFMI, kg/m^2^), appendicular skeletal muscle mass (ASMM, kg), skeletal muscle index (SMI, kg/m^2^), total body water (TBW, kg), extracellular water (ECW, kg), intracellular water (ICW, kg), hydration levels (Hydragram^®^, %TBW/FFM), nutrition-related impedance markers (Nutrigram^®^, mg/24 h/m), reactance (Xc, Ω/m), and resistance (Rz, Ω/m). Height was measured using a stadiometer (Holtain Limited, Crymych, UK), and body weight was measured using a calibrated scale accurate to 0.1 kg (SECA 665, Hamburg, Germany); patient-reported values were used when direct measurements were not feasible. BIVA measurements were standardized by sex and age using reference data from healthy Italian adults. (13) PhA was calculated as arctan (Xc/Rz) × (180°/π). Standardized PhA (SPhA) was calculated using age- and sex-specific reference data. Device calibration was performed daily using the standard control circuit supplied by the manufacturer, with a known impedance resistance (Rz) = 380 ohm; reactance (Xc) = 45 ohm.

#### 2.3.2. Ultrasound Muscle Measurement

Muscle ultrasound of the rectus femoris in the quadriceps was performed in all participants using a multifrequency linear transducer (10–12 MHz; Mindray^®^ Z60, Mindray Bio-Medical Electronics Co., Ltd., Shenzhen, China) with the patient in a supine position. Assessments followed the standardized Nutritional Ultrasound^®^ protocol [15]. All examinations were conducted by a trained dietitian, who positioned the probe perpendicular to the longitudinal and transverse axes of the QRF to measure the rectus femoris cross-sectional area (RF-CSA), circumference (RF-CIR), axes (X-axis and Y-axis), and leg subcutaneous fat (L-SAT). For each variable, three consecutive measurements were recorded, and the average value was used for analysis. Abdominal adipose tissue was assessed at the midpoint between the xiphoid process and the umbilicus, including measurements of total subcutaneous abdominal fat (T-SAT), superficial subcutaneous abdominal fat (S-SAT), and visceral adipose tissue (VAT).

#### 2.3.3. Functional Measurement

Handgrip strength (HGS) was measured using a Jamar^®^ hydraulic dynamometer (Asimow Engineering Co., Los Angeles, CA, USA) with participants seated and the dominant arm flexed at 90°. Three attempts were averaged, and the mean value was used for analysis. Physical performance was assessed via the Timed Up and Go test, recording the time to stand up, walk 3 m, turn, and return to the chair. Cut-off points for low muscle strength were defined according to the European Working Group on Sarcopenia in Older People (EWGSOP) consensus [16].

### 2.4. Statistical Analysis

Quantitative variables were expressed as mean ± SD. Normality was assessed using the Shapiro–Wilk test. Comparisons between qualitative variables were conducted using the chi-square test, with Fisher’s exact correction applied when appropriate. Quantitative variables were compared using Student’s *t*-test for normally distributed data and non-parametric tests (Mann–Whitney or Kruskal–Wallis) when normality was not met.

The discriminatory ability of phase angle (PhA) to distinguish between survivors and non-survivors was assessed using receiver operating characteristic (ROC) curves and the corresponding area under the curve (AUC), plotting sensitivity against specificity. ROC curve analyses were performed using non-parametric methods. The AUC and its 95% confidence interval were estimated using the DeLong method.

Logistic regression models were used to evaluate the association between mortality and malnutrition, defined according to GLIM criteria.

Given the limited number of mortality events, multivariable analyses were restricted to highly parsimonious models to minimize overfitting. Baseline GLIM-defined malnutrition was evaluated as the primary exposure, while additional covariates were explored only in sensitivity analyses.

Data analysis was conducted using JAMOVI (version 2.5.44, The Jamovi Project, Sydney, Australia). Statistical significance was defined as *p* < 0.05.

## 3. Results

### 3.1. General Baseline Characteristics of the Patients Included in the Study (Table 1)

A total of 52 patients were included in the study, of whom 57.7% were women (n = 30) and 42.3% were men (n = 22). The mean age of the cohort was 52.5 ± 17.5 years, with no significant differences between women and men. The distribution of leukemia types was as follows: 26.9% acute lymphoblastic leukemia (ALL), 67.3% acute myeloid leukemia (AML), and 5.8% undifferentiated leukemia. Regarding treatment, 84.6% of patients initiated intensive chemotherapy, 11.5% received hypomethylating agents, and 3.8% were treated with BCL2 inhibitors.

**Table 1:** Baseline characteristics of the population included in the study divided by sex.

	All	Women	Men	*p* Value
Variables	n = 52	n = 30 (57.7%)	n = 22 (42.3%)	
Anthropometric and demographic variables				
Age, years	52.5 (17.5)	56.2 (17.8)	53.5 (17.4)	0.577
Weight, kg	72,8 (13.2)	70.4 (14.2)	76.1 (12.9)	0.14
Body-mass index, kg/m^2^	26.4 (5.03)	27.3 (5.74)	25.2 (3.67)	0.15
Brachial circumference, cm	28.9 (3.66)	29.2 (4.02)	28.4 (3.13)	0.701
BIVA variables				
PhA, (°)	5.12 (0.97)	4.98 (0.89)	5.31 (1.08)	0.237
SPhA	−1.13 (0.90)	−0.93 (0.84)	−1.20 (0.93)	0.055
BCM, kg	25.4 (6.56)	22.2 (4.29)	29.8 (6.68)	<0.001 ***
FFM, kg	52.1 (9.48)	46.4 (5.74)	59.9 (7.99)	<0.001 ***
FM, kg	20.8 (10.2)	24 (11.1)	16.4 (7.02)	0.007 **
ASMM, kg	20.1 (4.68)	17.5 (3.23)	23.7 (3.89)	<0.001 ***
SMI, kg/m^2^	9.03 (1.92)	8.14 (1.79)	10.3 (1.35)	<0.001 ***
Na/K	1.16 (0.29)	1.09 (0.29)	1.26 (0.25)	0.04 *
Hydragram^®^, %	75.2 (3.97)	75.2 (4.30)	75.1 (3.58)	0.962
Nutrigram^®^, mg/24 h/m	730 (192)	663 (124)	821 (230)	0.002 **
Nutritional Ultrasound				
RF-CSA, cm^2^	4.29 (1.49)	3.67 (1.04)	5.10 (1.61)	<0.001 ***
RF-X-axis, cm	3.55 (0.626)	3.30 (0.606)	3.88 (0.49)	<0.001 ***
RF-Y-axis, cm	1.29 (0.43)	1.24 (0.49)	1.36 (0.35)	0.340
L-SAT, cm	1.10 (0.61)	1.46 (0.53)	0.59 (0.20)	<0.001 ***
T-SAT, cm	1.65 (0.86)	1.83 (0.88)	1.43 (0.80)	0.100
S-SAT, cm	0.78 (0.44)	0.95 (0.49)	0.56 (0.25)	0.001 **
VAT, cm	0.64 (0.36)	0.67 (0.38)	0.62 (0.35)	0.653
Functional parameters				
Handgrip strength, kg	25.4 (11.3)	19.4 (5.81)	33.5 (12.1)	<0.001 ***
Test Up and Go, s	8.37 (2.16)	8.98 (2.42)	7.65 (1.58)	0.061

Data are expressed as mean ± standard deviation (SD) for continuous variables and as number and percentage [n (%)] for categorical variables. * *p* < 0.05, ** *p* < 0.01, *** *p* < 0.001. Abbreviations: BCM: body cell mass; BIVA: bioelectrical impedance vectorial analysis; FM: fat mass; FFM: fat-free mass; PA: phase angle; RF-CSA: rectus femoris cross-sectional area; SAT: subcutaneous adipose fat of leg (L), superficial (S) and total (T) abdominal; SMI: skeletal muscle index; SPA: standardized phase angle.

Baseline digestive symptoms were frequent, with 53.8% reporting hyporexia, 25% nausea, 21.2% constipation, 17.3% diarrhea, 3.8% dysphagia, and 17.3% presenting edema.

Anthropometric parameters, including body weight, body mass index, and brachial circumference, showed no sex-related differences.

Regarding BIVA-derived measurements, phase angle and standardized phase angle did not differ significantly between groups. In contrast, men presented higher values of body cell mass, fat-free mass, appendicular skeletal muscle mass, and skeletal muscle index (all *p* < 0.001), together with higher Nutrigram^®^ values (*p* = 0.002). Women showed greater fat mass (*p* = 0.007). Hydration parameters, including Na/K ratio and Hydragram^®^ percentages, were similar between sexes.

Nutritional ultrasound revealed significantly larger rectus femoris cross-sectional area and X-axis dimensions in men (both *p* < 0.001), while subcutaneous leg fat was higher in women (*p* < 0.001). Visceral adipose tissue and several other ultrasound-derived parameters showed no significant differences.

Functional assessments indicated markedly higher handgrip strength values in men compared to women (*p* < 0.001), while Timed Up and Go test results did not differ significantly.

At baseline, 65.4% of the cohort met the GLIM criteria for malnutrition.

At the 12-month follow-up, overall mortality in the cohort was 25%. No significant sex differences were found in GLIM-defined malnutrition prevalence (*p* = 0.62), nor in its association with 12-month mortality (*p* = 0.61).

Demographic characteristics, anthropometric measurements, functional test results, and patients’ measurements by survival or not are shown in Supplementary Table S1.

### 3.2. Changes from Baseline to Three-Month Follow-Up (Table 2)

At the 3-month follow-up—conducted after completion of initial treatment and prior to hematopoietic stem cell transplantation—patients showed significant declines in several anthropometric and body composition parameters.

**Table 2:** Changes in nutritional status after nutritional intervention in the patients included in the study (n= 52), 3 months after treatment.

	Baseline Mean (SD)	After Mean (SD)	*p* Value	Change
Anthropometric and demographic variables				
Weight, kg	72.8 (13.2)	68.3 (14.2)	<0.001 ***	−4.51
Body-mass index, kg/m^2^	26.4 (5.03)	24.7 (5.16)	<0.001 ***	−1.80
Brachial circumference, cm	28.9 (3.66)	27.8 (5.00)	0.022 **	−1.1
BIVA variables				
PhA, (°)	5.12 (0.97)	4.57 (1.08)	<0.001 ***	−0.55
SPhA	−1.13 (0.90)	−1.65 (1.07)	<0.001 ***	−0.52
BCM, kg	25.4 (6.56)	22.25 (6.92)	<0.001 ***	−3.15
FFM, kg	52.1 (9.48)	48.64 (9.12)	<0.001 ***	−3.76
FM, kg	20.8 (10.2)	19.6 (10.4)	0.590	−1.2
ASMM, kg	20.1 (4.68)	18.44 (4.59)	<0.001 ***	−1.66
SMI, kg/m^2^	9.03 (1.92)	8.33 (1.60)	0.002 **	−0.70
Na/K	1.16 (0.29)	1.13 (0.42)	0.603	0.04
Hydragram^®^, %	75.2 (3.97)	74.6 (2.78)	0.355	−0.60
Nutrigram^®^, mg/24 h/m	730 (192)	639 (169)	0.002 **	−91.0
Nutritional Ultrasound				
RF-CSA, cm^2^	4.29 (1.45)	3.93 (1.46)	0.011 **	−0.36
RF-X-axis, cm	3.55 (0.63)	3.59 (0.52)	0.556	0.04
RF-Y-axis, cm	1.29 (0.43)	1.16 (0.36)	0.019 **	−0.13
L-SAT, cm	1.10 (0.61)	0.90 (0.49)	0.06	0.20
T-SAT, cm	1.65 (0.86)	1.73 (0.92)	0.633	0.08
S-SAT, cm	0.78 (0.44)	0.77 (0.43)	0.296	−0.01
VAT, cm	0.64 (0.36)	0.67 (0.40)	0.404	0.03
Functional parameters				
Handgrip strength, kg	25.4 (11.3)	24.33 (11.6)	0.235	−1.07
Test Up and Go, s	8.37 (2.16)	7.42 (2.69)	0.069	0.95

Data are expressed as mean ± standard deviation (SD) for continuous variables. ** *p* < 0.01, *** *p* < 0.001. Abbreviations: BCM: body cell mass; BIVA: bioelectrical impedance vectorial analysis; FM: fat mass; FFM: fat-free mass; PA: phase angle; RF-CSA: rectus femoris cross-sectional area; SAT: subcutaneous adipose fat of leg (L), superficial (S), and total (T) abdominal; SMI: skeletal muscle index; SPA: standardized phase angle.

Mean body weight decreased by 4.51 kg, accompanied by a reduction in BMI (−1.80 kg/m^2^; *p* < 0.001) and brachial circumference (−1.1 cm; *p* = 0.022). BIVA-derived metrics demonstrated substantial deterioration in cellularity and muscle mass, with significant reductions in phase angle (−0.55°, *p* < 0.001) and standardized phase angle (−0.524, *p* < 0.001). Correspondingly, body cell mass (−3.15 kg), fat-free mass (−3.76 kg), appendicular skeletal muscle mass (−1.66 kg), and skeletal muscle index (−0.70 kg/m^2^) all declined significantly (all *p* < 0.01). No significant change was observed in fat mass.

Fluid status was monitored using Hydragram^®^ and clinical evaluation of edema. No significant changes were observed between timepoints. The Na/K ratio did not show a significant decrease. Nutrigram^®^ values declined (−91 mg/24 h/m; *p* = 0.002).

Muscle ultrasound findings reflected similar patterns of muscle deterioration, with a significant reduction in rectus femoris cross-sectional area (−0.36 cm^2^; *p* = 0.011) and in the Y-axis diameter (−0.13 cm; *p* = 0.019). Subcutaneous fat parameters did not significantly change, although a trend toward reduced leg subcutaneous fat was observed. Visceral adipose tissue remained stable.

Regarding functional outcomes, handgrip strength showed a nonsignificant decline (−1.07 kg; *p* = 0.235), while performance in the Timed Up and Go test demonstrated a trend toward improvement, although without reaching statistical significance.

Overall, the 3-month assessment revealed a marked deterioration in muscle mass, cellularity, and several nutritional biomarkers, despite relative preservation of functional capacity.

### 3.3. Twelve-Month Mortality

Twelve-month mortality was analyzed according to baseline malnutrition status, defined using GLIM criteria. Logistic regression analysis demonstrated that baseline GLIM-defined malnutrition was significantly associated with a higher likelihood of death at 12 months. In the multivariable model, baseline GLIM-defined malnutrition remained significantly associated with 12-month mortality (OR = 6.88; 95% CI: 1.17–40.38; *p* = 0.033).

Changes in phase angle (ΔPhA) between baseline and the 3-month reassessment were also evaluated for their association with 12-month mortality. Patients who died during follow-up experienced a greater decline in PhA compared with survivors, suggesting progressive deterioration in cellular integrity and metabolic reserves during initial treatment phases.

**Figure 1:**
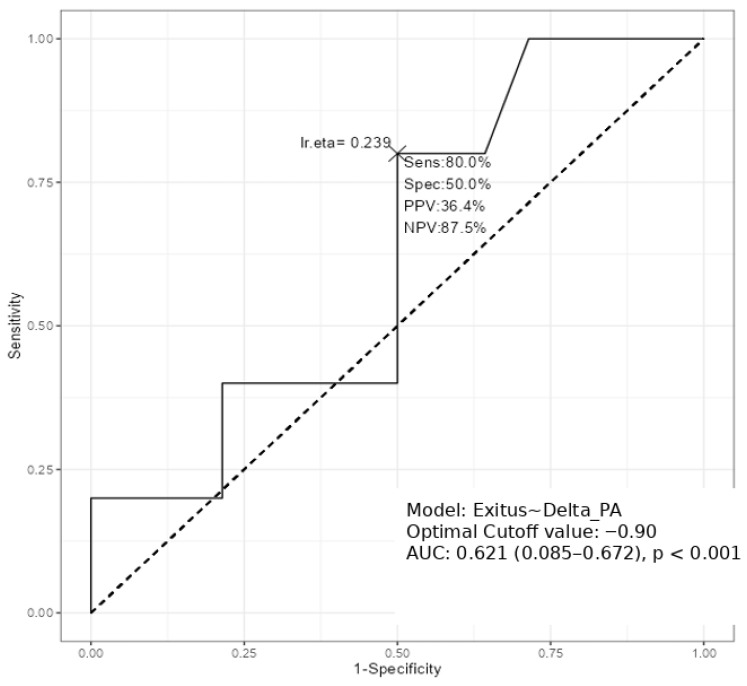
ROC Analysis of the Discriminatory Ability of Phase Angle Change (ΔPhA) for 12-Month Mortality in Acute Leukemia. The expression “Exitus ~ Delta_PA” reflects the model used in the analysis. Abbreviations: ROC = Receiver Operating Characteristic; AUC = Area Under the Curve; Delta_PA = change in Phase Angle.

Receiver operating characteristic (ROC) curve analysis was used as an exploratory discrimination analysis to assess the ability of ΔPhA to distinguish between survivors and non-survivors, yielding an AUC of 0.62 (95% CI: 0.08–0.67; *p* < 0.001). A PhA reduction of −0.90° was identified as the optimal cut-off in this exploratory analysis, with a sensitivity of 80.0% and a specificity of 50.0% for 12-month mortality. (Figure 1) When ΔPhA was included as a dichotomized variable (≥0.90° vs. <0.90°) in a multivariable logistic regression model for 12-month mortality, the association did not reach statistical significance (OR = 1.50, 95% CI: 0.19–11.93; *p* = 0.702). However, the direction of the effect remained consistent with the univariate analysis and the ROC curve findings. The lack of statistical significance is likely related to the modest sample size and limited number of events.

## 4. Discussion

In this prospective cohort of patients with acute leukemia, we observed a markedly high prevalence of malnutrition at baseline (65.4% according to GLIM criteria), substantially exceeding the prevalence reported in previous studies, which typically ranges between 15 and 26.5% in acute leukemia cohorts [4,13,17] and 40–80% among cancer patients overall [18]. Unlike the Iranian cohort assessed before transplantation, which included younger patients and reported a 26.5% prevalence of GLIM-defined malnutrition with no significant association with short-term mortality [19], our sample displayed both a higher burden of malnutrition and a clear association with 12-month mortality. This discrepancy may be partly explained by differences in age, disease severity, and timing of the nutritional evaluation. Furthermore, studies using Subjective Global Assessment (SGA) in acute leukemia populations have similarly reported high malnutrition rates (up to 64.7%), noting increased chemotherapy toxicity, higher minimal residual disease, and poorer survival in severely malnourished patients, thereby reinforcing the prognostic relevance of early nutritional deficits [3]. After the initial chemotherapy cycle, the 3-month reassessment revealed substantial deterioration in multiple nutritional domains. Significant reductions were observed in phase angle (PhA), standardized PhA, fat-free mass, skeletal muscle mass, body cell mass, and rectus femoris muscle parameters. Overall, the 3-month assessment revealed a marked deterioration in muscle mass, cellularity, and several nutritional biomarkers, despite relative preservation of functional capacity. These findings are consistent with prior evidence showing that induction or intensive chemotherapy induces profound catabolic effects, leading to muscle wasting and impaired cellular integrity even when body weight or BMI appear relatively preserved [20,21,22]. Such discordance reinforces the limitations of BMI as a standalone indicator of nutritional decline and the value of incorporating morphofunctional tools—including BIVA, muscle ultrasound, and handgrip strength—into routine assessment. One of the key findings of our study is the prognostic relevance of both baseline GLIM-defined malnutrition and early declines in PhA. Baseline malnutrition remained significantly associated with 12-month mortality (OR 6.88), supporting the clinical utility of GLIM for risk stratification in acute leukemia. Our results align with evidence indicating that GLIM and SGA, particularly when combined with objective muscle strength measures, effectively identify high-risk oncology patients [23,24]. In addition, deterioration in phase angle (ΔPhA) over the first three months was significantly associated with 12-month mortality. These results are consistent with growing evidence positioning PhA as a sensitive indicator of cellular integrity, hydration balance, and overall nutritional status, with strong associations with morbidity and mortality in cancer and transplant populations [8,20,21,25,26,27]. Although ΔPhA was significantly associated with mortality in our cohort, its discriminative performance was modest, and the proposed cut-off should be considered exploratory further validation is necessary before clinical implementation.

Previous research has shown that patients with low PhA or declining PhA experience greater loss of body cell mass, muscle mass, and intracellular water following antineoplastic treatment, supporting the biological plausibility of PhA as a dynamic prognostic marker [28]. While prior studies have reported sex-specific PhA cut-offs for mortality (<3.8° for females, <5.4° for males) in patients admitted with haematologic cancer [10], our study suggests that changes in PhA (ΔPhA), rather than an absolute threshold, were more strongly associated with 12-month mortality. Interestingly, baseline PhA was not significantly associated with 12-month mortality in our cohort, despite previous studies supporting its prognostic value in oncology. This discrepancy may reflect the limited number of events in a relatively small sample, as well as the high metabolic burden of acute leukemia and its treatment. In this context, dynamic PhA decline may more accurately reflect progressive cellular deterioration and be more strongly associated with adverse outcomes. This underscores the importance of monitoring PhA longitudinally, as dynamic declines may capture early metabolic deterioration more sensitively than single timepoint measurements. Although ΔPhA did not remain statistically significant in logistic regression analysis, the consistency in effect direction and its discriminatory capacity in ROC analysis suggest a potential prognostic role. The wide confidence intervals observed reflect limited statistical power due to the small sample size and low number of mortality events. The limited number of events constrained the complexity of multivariable models. Therefore, results from adjusted analyses should be interpreted as exploratory. In acute leukemia, where early metabolic deterioration may be more relevant than baseline status, dynamic changes in PhA could represent a clinically meaningful marker that requires confirmation in a larger cohort. The multidimensional changes observed after three months—particularly the marked reduction in fat-free mass, skeletal muscle mass, BCM, and PhA—reinforce prior findings that chemotherapy exerts profound negative effects on body composition and nutritional status in patients with leukemia [19]. These catabolic changes highlight the limitations of relying solely on BMI, which often fails to detect early muscle loss or cellular deterioration in this population [22]. In this context, the integration of BIVA, muscle ultrasound, and functional testing provides a more sensitive and comprehensive approach to nutritional assessment. Given the consistent association between worsening PhA and higher mortality risk, our results strongly support the implementation of early and proactive nutritional intervention, ideally initiated before or at the start of chemotherapy, to mitigate treatment-related deterioration. In addition, we explored sex-related differences in nutritional status and outcomes. Although several baseline morphofunctional parameters differed between sexes, GLIM-defined malnutrition was equally prevalent in men and women, and its prognostic value remained consistent across sexes. These findings suggest that the impact of early nutritional impairment on survival in acute leukemia may be independent of sex-specific body composition patterns.

The strengths of our study include its prospective design, the use of multiple complementary methods for nutritional evaluation (GLIM, BIVA, muscle ultrasound, and functional tests), and the longitudinal follow-up capturing clinically relevant outcomes up to 12 months. Additionally, the reassessment performed before hematopoietic stem cell transplantation offers valuable insight into the dynamic impact of initial treatment on body composition and survival-related outcomes. However, several limitations must be acknowledged. First, the sample size was modest, which may limit the statistical power for subgroup analyses. Second, although a quantitative dietary intake assessment was not performed, data on intake levels and gastrointestinal symptoms were collected. Inflammatory and biochemical markers—including albumin, total proteins, C-reactive protein (CRP), and lymphocyte count—were also recorded but not analyzed in the present study, which limits the capacity to explore potential mechanisms linking nutritional deterioration and prognosis. Future research should consider integrating biochemical profiling with morphofunctional tools to enhance nutritional risk stratification, as supported by recent literature [29,30]. Finally, although changes in PhA showed an association with mortality, causality cannot be established, and interventional trials are needed to determine whether targeted nutritional strategies can improve PhA and survival. Although morphofunctional assessment tools such as BIVA and muscle ultrasound require specific equipment and trained personnel, all measurements in this study were performed by experienced professionals using standardized protocols. Nevertheless, the availability of such expertise may be limited outside specialized hematology or stem cell units, which could affect the generalizability of our findings.

## 5. Conclusions

In summary, our study demonstrates that malnutrition—identified early using GLIM criteria and functional muscle assessments—and declines in phase angle during initial treatment are strongly associated with 12-month mortality in patients with acute leukemia. These findings underscore the importance of comprehensive nutritional evaluation and support the incorporation of BIVA-derived parameters, particularly PhA and SPhA, into routine clinical practice for risk stratification. Although we did not evaluate the effects of nutritional intervention directly, the association between ΔPhA and mortality suggests that patients experiencing early declines—regardless of baseline PhA—may benefit from intensified nutritional monitoring and support. This hypothesis should be explored in future interventional studies to determine whether early nutritional strategies can mitigate adverse outcomes in this vulnerable population.

## Data Availability

The data that support the findings of this study are available from the corresponding author upon reasonable request. Due to ethical restrictions and patient confidentiality, the data are not publicly available.

## Supplementary Materials

The following supporting information can be downloaded at https://www.mdpi.com/article/10.3390/nu18030374/s1. Table S1: Baseline demographic, anthropometric, body composition, and functional characteristics according to 12-month survival status. Figure S1: Flow chart diagram.

## Abbreviations

The following abbreviations are used in this manuscript:

ALL	Acute Lymphoblastic Leukemia
AML	Acute Myeloid Leukemia
BIA	Bioelectrical Impedance Analysis
BIVA	Bioelectrical Impedance Vector Analysis
BCM	Body Cell Mass
CSA	Cross-Sectional Area
GLIM	Global Leadership Initiative on Malnutrition
HGS	Handgrip Strength
PhA	Phase Angle
ROC	Receiver Operating Characteristic
SGA	Subjective Global Assessment
