# Supplementary material for: Bioelectrical Impedance and GLIM Criteria Identify Early Nutritional Deterioration and Mortality in Acute Leukemia Patients Undergoing Chemotherapy
Source: Nutrients. 2026 Jan 23;18(3):374. doi: 10.3390/nu18030374 (PMC12899684; doi:10.3390/nu18030374)
Supplement: Supplementary file 1 [file nutrients-18-00374-s001.zip › nutrients-4073847-supplementary.pdf]

Supplementary Figure S1: Flow chart diagram

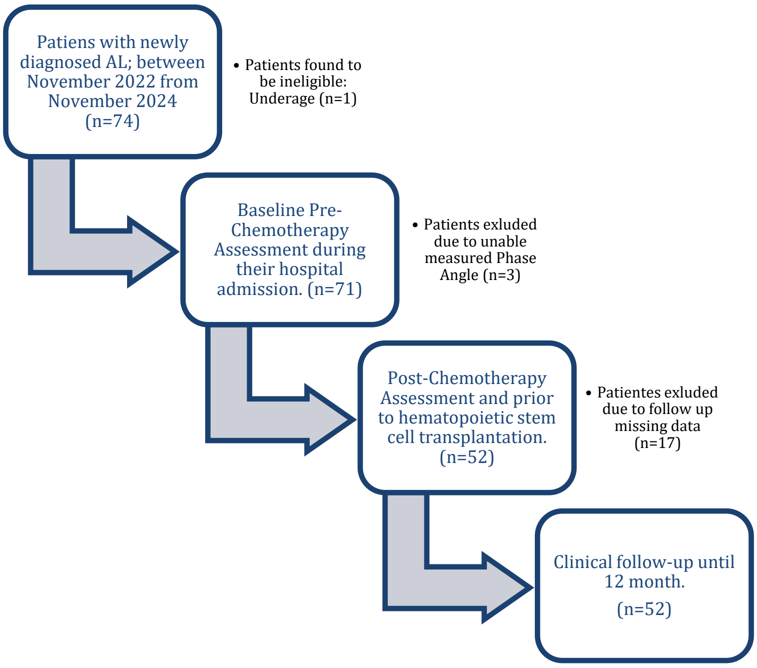

Supplementary Table S1. Baseline demographic, anthropometric, body composition, and functional characteristics according to 12-month survival status

	All	Alive	Deceased	P value
Variables	n=52	n=39 (75%)	n=13 (25%)	
**Anthropometric and demographic variables**				
Age, years	52.50 (17.5)	50.40 (18.9)	57.60 (16.9)	0.11
Weight, kg	72.80 (13.2)	73.31 (13.91)	71.07 (14.25)	0.88
Body-mass index, kg/m2	26.40 (5.03)	26.20 (4.56)	26.80 (5.74)	0.40
Brachial circumference,cm	28.90 (3.66)	28.41 (3.06)	29.14 (5.07)	0.63
**BIVA variables**				
PA, (º)	5.12 (0.97)	5.15 (0.98)	5.00 (0.99)	0.17
SPA	-1.13 (0.90)	-1.07 (0.96)	-1.15 (1.01)	0.36
BCM, kg	25.40 (6.56)	25.60 (6.29)	24.60 (5.70)	0.26
FFM, kg	52.10 (9.48)	51.70 (7.75)	52.50 (9.85)	0.59
FM, kg	20.80 (10.2)	20.6 (9.5)	21.10 (10.7)	0.46
ASMM, kg	20.10 (4.68)	20.8 (4.95)	19.70 (4.15)	0.49
SMI, kg/m2	9.03 (1.92)	9.31 (1.97)	8.98 (1.88)	0.62
Na/K	1.16 (0.29)	1.17 (0.21)	1.16 (0.28)	0.53
Hydragram®, %	75.20 (3.97)	75.20 (3.86)	75.10 (4.02)	0.66
Nutrigram®, mg/24h/m	730 (192)	732 (217)	724 (202)	0.94
**Nutritional Ultrasound**				
RF-CSA, cm2	4.29 (1.49)	4.51 (1.16)	4.13 (1.73)	0.47
RF-X-axis, cm	3.55 (0.63)	3.57 (0.47)	3.50 (0.65)	0.96
RF-Y-axis, cm	1.29 (0.43)	1.30 (0.30)	1.21 (0.33)	0.43
L-SAT, cm	1.10 (0.61)	1.22 (0.61)	1.07 (0.65)	0.88
T-SAT, cm	1.65 (0.86)	1.68 (0.88)	1.64 (0.82)	0.66
S-SAT, cm	0.78 (0.44)	0.80 (0.46)	0.77 (0.43)	0.32
VAT, cm	0.64 (0.36)	0.65 (0.36)	0.64 (0.38)	0.70
**Functional parameters**				
Handgrip strength, kg	25.40 (11.3)	25.70 (10.43)	23.40 (12.70)	0.43
Test Up and Go, s	8.37 (2.16)	7.93 (1.10)	8.50 (1.91)	0.12

Abbreviations: BCM: body cell mass; BIVA: bioelectrical impedance vectorial analysis; FM: fat mass; FFM: fat-free mass; PA: phase angle; RF-CSA: rectus femoris cross-sectional area; SAT: subcutaneous adipose fat of leg (L), superficial (S) and total (T) abdominal; SMI: skeletal muscle index; SPA: standardized phase angle
